# Control of viral infections by epigenetic-targeted therapy

**DOI:** 10.1186/s13148-019-0654-9

**Published:** 2019-03-27

**Authors:** Zeina Nehme, Sébastien Pasquereau, Georges Herbein

**Affiliations:** 1 Department Pathogens & Inflammation-EPILAB, UPRES EA4266, University of Franche-Comté, University of Bourgogne Franche-Comté, 16 route de Gray, F-25030 Besançon cedex, France; 2 Université Libanaise, Beirut, Lebanon; 3 Department of Virology, CHRU Besancon, F-25030 Besançon, France

**Keywords:** Virus, HIV, HCMV, Epigenetics, Treatment, Cancer

## Abstract

Epigenetics is defined as the science that studies the modifications of gene expression that are not owed to mutations or changes in the genetic sequence. Recently, strong evidences are pinpointing toward a solid interplay between such epigenetic alterations and the outcome of human cytomegalovirus (HCMV) infection. Guided by the previous possibly promising experimental trials of human immunodeficiency virus (HIV) epigenetic reprogramming, the latter is paving the road toward two major approaches to control viral gene expression or latency. Reactivating HCMV from the latent phase (“shock and kill” paradigm) or alternatively repressing the virus lytic and reactivation phases (“block and lock” paradigm) by epigenetic-targeted therapy represent encouraging options to overcome latency and viral shedding or otherwise replication and infectivity, which could lead eventually to control the infection and its complications. Not limited to HIV and HCMV, this concept is similarly studied in the context of hepatitis B and C virus, herpes simplex virus, and Epstein-Barr virus. Therefore, epigenetic manipulations stand as a pioneering research area in modern biology and could constitute a curative methodology by potentially consenting the development of broad-spectrum antivirals to control viral infections in vivo.

## Background

Since its emergence for the first time in 1940, the epigenetic field is witnessing a continuous surge over the last decades [1]. Although the epigenetic term is well thought out to be a large umbrella under which falls concepts related to development, heredity, and evolution [2], recent technical advancements have narrowed the term’s definition in the standpoint of molecular biology [3]. Hence, epigenetics could be defined as “the study of heritable changes in gene expression that are not due to changes in DNA sequence” [4]. Recent numerous literature is showing a correlation between epigenetic modifications and a wide array of human diseases including—but not limited to—cancer, neurological and psychiatric disorders (Alzheimer’s disease, schizophrenia), autoimmune disorders (rheumatoid arthritis, systemic lupus erythematosus), and others [5–7]. However, this association was converted and extended to the clinical level first in the cancerology field with the FDA-approved DNA methyltransferase (DNMT) inhibitors (azacytidine, decitabine) and histone deacetylase (HDAC) inhibitors (vorinostat, romidepsin, belinostat, panobinostat) [8]. In fact, in addition to DNMT and HDAC, the epigenetic machinery entails composite complexes that each of which could constitute a valuable target for the development of potential new epigenetic antiviral drugs [9]. This review examines and discusses the involvement and the role of various epigenetic players throughout the different viral life cycle stages and highlights their potential implications in the clinical management of several viral infections, especially human immunodeficiency virus (HIV) and human cytomegalovirus (HCMV), in addition to hepatitis viruses, herpes simplex virus-1 (HSV-1), and Epstein-Barr virus (EBV).

## HIV and epigenetics, a leading example as a proof of concept

**Fig. 1:**
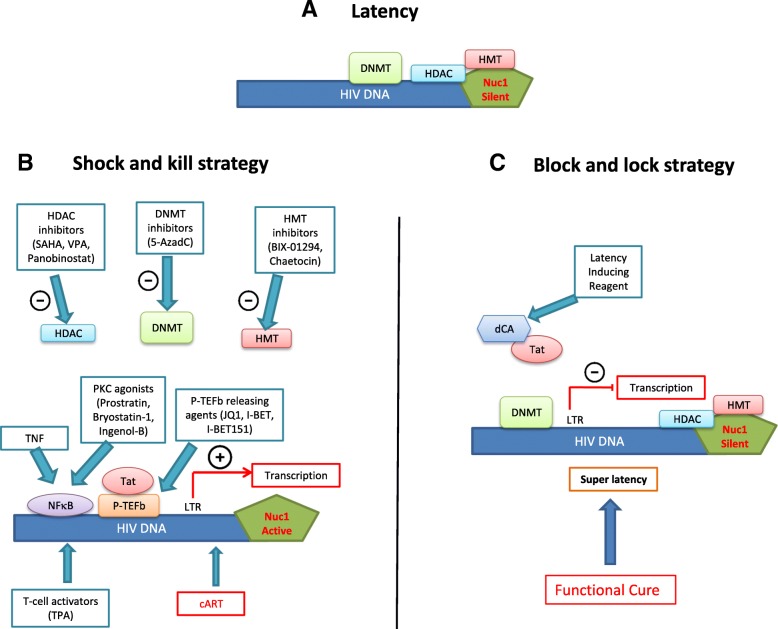
Epigenetic manipulation to eradicate HIV: “shock and kill” or “block and lock”? **a** Latent HIV provirus-established reservoirs in infected resting CD4+ memory T cells and myeloid cells are not eliminated by cART and are thus prone to be reactivated after cART discontinuation. One strategy to eliminate those reservoirs is the “shock and kill” therapy. **b** Shock-inducer agents like histone deacetylase (HDAC), DNA, or histone methyltransferase (DNMT and HMT respectively) inhibitors used alone or in combination with other players (PKC agonists, P-TEFb releasing agents, TNF, TPA) could reverse latency through the removal of repressive silencing marks imposed on the nucleosome Nuc-1 or the DNA. This purges the viral reservoirs and leads eventually to the clearance of virus-harboring cells along with cART. On the other hand (**c**), blocking Tat, a viral protein indispensable for the recruitment of transcriptional factors like the positive transcription elongation factor B (P-TEFb), by a latency inducing reagent such as dCA reduces viral transcription and locks the HIV promoter in a super-latency state resistant to any reactivation stimuli leading potentially to a functional cure

Since the introduction of combination antiretroviral therapy (cART), survival and quality of life among HIV-infected patients significantly improved [10], with a more favorable outcome with therapy initiation in the setting of early asymptomatic infection [11]. This shifted HIV conception from a non-curable devastating fatal illness to a possibly manageable chronic one. However, cART is yet not the ideal road map for HIV management, as physical and psychological burden are still imposed by this therapy [12, 13], leading sometimes to a reduced compliance or even discontinuation [14]. Markedly, a major limitation associated with cART cessation is viral rebound [15]. This is due to the presence of HIV reservoirs, mainly in the latently infected resting CD4+ memory T cells and myeloid cells such as macrophages and microglia, that are difficult to be targeted by cART or immune effector mechanisms [16–18]. Interestingly, the integrated provirus in those cells is subjected to transcriptional silencing by host chromatin-modifying enzymes, comprising deacetylases, methyltransferases, and others [19]. This paved the road to the emergence of two new epigenetic therapeutic approaches, namely the “shock and kill” and the “block and lock” strategies [20–23]. Here, we present general points about these two anti-HIV therapeutic strategies (Fig. 1, Table 1).

**Table 1:** Functional outcomes of epigenetic regulation in viral infections

Target class	Target	Inhibitor	Virus studied	Functional outcome
HDM	JMJD2	ML324	HCMV	Repression of viral IE gene expression and viral yields [95, 97]
		DMOG	HCMV	Decrease in the expression of HCMV IE genes UL37, UL72, and US3 [97]
		DMOG and ML342	HSV-1	Significant decreased in the viral titers in trigeminal ganglia of HSV-1 latently infected mice [97]
	LSD1	OG-L002	HCMV	Repression of HCMV IE expression [96]
		TCP	HCMV	Decrease in the expression of HCMV IE genes UL37, UL72, and US3 [97]
			HSV-1	Repression of HSV IE gene expression and genome replication in vivo
				Decrease in the severity of a virus-induced encephalitis and corneal blindness in mouse models
				Blockage of viral reactivation in trigeminal ganglia
			Adenovirus	Reduction in E1A gene expression [96]
HDAC	Class II HDAC4	MC1568	HCMV	Induction of transient expression of the viral lytic IE antigens without full virus reactivation [104]
	Histone deacetylase	Sodium butyrate	HSV-1	Production of infectious progeny in quiescently infected cells [154]
			EBV, KSHV	Latency reversal [179]
		TSA, SAHA, VPA, and suberoylanilide hydroxamic acid	HSV-1	Reduction in the number of HSV-1 genomes that initiate replication [164]
		TSA, VPA	HBV	Increase in HBV transcripts
				Cytoplasmic accumulation of HBV replicative intermediates
				Increase in secreted HBV viral particles [128]
		SAHA	HCV	Suppression of HCV replication without affecting cell viability [135]
	Histone deacetylase 3	RGFP966	HCV	Reduction of viral replication in Huh7 cells and an in vivo model of humanized transgenic mice [141]
	Histone deacetylase 6	Tubastatin A	HCV	Suppression of HCV replication in HepG2 cells [137]
	Pan-histone deacetylase	SAHA + TPA	HIV	Purging HIV-1 proviruses in HIV-1 latently infected cells via ERK and AP-1 pathways [26]
HMT	EZH2	(DZnep)	HCMV	Significant activation of the lytic transcriptional program [85]
		GSK126 and GSK343	HSV-1	Blockage of lytic viral replication in latently infected ganglion explant model [169]
	Suv39H	Chaetocin	HIV	HIV-1 recovery in resting CD4^+^ T cells [36]
	G9a	BIX-01294	HIV	HIV-1 recovery in resting CD4^+^ T cells [36]
HAT	p300/CBP	C646	HBV	Reduction in HBV transcription in a dose-dependent manner [111]
DNMT	DNMT	Azacitidine	HBV	Tumor growth inhibition and decreased aggressiveness in vitro and in vivo [123]
			HCV	Inhibition of HCV infection [150]
Viral protein	Tat (transactivator of transcription)	Didehydro-cortistatin A (dCA)	HIV	Reduction of residual levels of viral transcription in several models of HIV latency
				Establishment of a nearly permanent state of latency [42]
				Suppression of viral rebound after ART interruption in HIV+ humanized BLT mice [43]

### Shock and kill strategy

This strategy is grounded on the concept that the latent HIV provirus could be switch on from latency (shock) into an active form prone to eradication (kill) through the humoral immune response, CD8+ T cells-mediated lysis, virus-induced apoptosis, or activation-induced cell death [24]. Several latency-reversing agents (LRA) or “shock” inducers have been proposed [25, 26] including histone deacetylase (HDAC), histone methyltransferase (HMT), and DNA methyltransferase (DNMT) inhibitors. Histone deacetylases family is composed of 18 enzymes that are gathered into four major groups: HDAC I–IV [27]. HDAC enzymes are responsible of removing acetyl groups from histones, which favors the formation of a compacted, transcriptionally repressed chromatin structure [28]. HDACs have gained an ascending attention after the FDA approval of HDACs inhibitors for cancer treatment [29], such as vorinostat or suberanilohydroxamic acid (SAHA) for the management of cutaneous T cell lymphoma [30] and panobinostat in relapsed multiple myeloma [31]. HDAC inhibitors like SAHA or DNMT inhibitors could be used alone to reactivate HIV gene expression along with efficient cART [32]. For instance, co-treatment with the HDAC inhibitor SAHA and the global T cell activator 12-O-tetradecanoylphorbol-13-acetate (TPA) revealed a significant synergistic effect on purging HIV-1 proviruses in HIV-1 latently infected cells [26]. Other activators of NF-kB such as prostratin have been also used in combination with HDAC inhibitors to reactivate HIV, these former players reactivating HIV in the absence of immune activation [33]. The concomitant use of protein kinase C (PKC) agonists (prostratin, bryostatin-1, and ingenol-B), which are known to activate NF-κB signaling pathway as well as the positive transcription elongation factor B (P-TEFb), used alone or in combination with P-TEFb-releasing agents (HMBA and Bromodomain and Extraterminal (BET) inhibitors JQ1, I-BET and I-BET151) leads to synergistic HIV reactivation from latency [34]. Moreover, sequential treatment with the DNMT inhibitor 5-aza-2′-deoxycytidine (5-AzadC) and HDAC inhibitors reactivates HIV-1 from latency [35]. In addition, it has been shown that the use of chaetocin and BIX-01294, specific inhibitors of HMT Suv39H1 and G9a respectively, resulted in HIV-1 recovery in resting CD4+ T cells in highly active antiretroviral therapy (HAART)-treated patients with undetectable viral load [36]. Another therapeutic approach could be considering tumor necrosis factor alpha (TNF)-based therapies, where combining HDAC inhibitors or HMT inhibitors with TNF, disrupts HIV-1 latency by triggering the activation of transcriptional activators like NF-κB and preventing the formation of heterochromatin, enhancing thus HIV-1 long terminal repeat (LTR) transcription and viral purge [37]. In fact, targeting several cellular proteins involved in the epigenetic control of viral gene expression usually amplifies HIV-1 reactivation. Although this approach is facing several hurdles, including—but not limited to—reactivating and possibly eliminating only a small subset of the latent HIV genome, it constitutes however one tactic that could be used in parallel to other approaches to achieve a fully effective cure [38].

### Block and lock strategy

In contrast to LRA, chemical agents could “block” the ongoing viremia during cART, by “locking” the HIV promoter in a super latency state resistant to reactivation stimuli. In fact, the “block and lock” strategy is emerging as a new approach to functionally cure HIV. Didehydro-cortistatin A (dCA), a specific and potent Tat inhibitor [39] has been studied in this context. Briefly, binding of the transactivator of transcription Tat to the HIV-1 mRNA results in the recruitment of indispensable transcriptional factors like the P-TEFb to induce sustained transcriptional elongation from the viral promoter LTR [40]. dCA binds specifically to the TAR-binding domain [41], reduces residual levels of viral transcription in several models of HIV latency, establishes a nearly permanent state of latency [42], and delays viral rebound after cART interruption in HIV+ humanized BLT mice [43]. This could be especially beneficial in cases of therapy non-compliance or short period discontinuation as dCA addition to cART regimens can limit the continual replenishment of the CD4+ T cell reservoir [44]. This could potentially stop the increased longevity and persistence of the latent viral reservoir observed in cART-treated patients by inhibiting new rounds of infection in CD4+ T cells [45]. Interestingly, as Tat is a HIV-specific viral protein with no cellular homolog, using dCA to “block and lock” HIV should not silence other regulatory pathways essential to fight other infections. In addition, Akt activation favors HIV-1 reactivation from resting CD4+ T cells and monocytes/macrophages, the two major HIV-1 cellular reservoirs [46–48]. Thus, Akt inhibitors, but also HIV protease inhibitors which display an anti-Akt activity [47], inhibit Akt activation in HIV-1 infected cells thereby favoring a “lock” stage, decreasing cell viability, and opening thus the door to the clearance of infected cells under Akt blockade. These results strongly encourage and open new insights to the possible addition of the “block and lock” approach as an additional potential therapeutic management strategy.

## Human cytomegalovirus and epigenetics

Human cytomegalovirus (HCMV) is a ubiquitous pathogen also denoted as human herpesvirus 5 (HHV5). It is a member of betaherpesvirinae, a subfamily of the *Herpesviridae* family [49]. HCMV infection is very common, as 40 to 95% of the population is seropositive [50]. However, the pathological outcomes depend on the host’s immune status, where infection in immunocompetent individuals rarely causes evident manifestations at the clinical level [51]. Conversely, HCMV infection significantly affects morbidity and mortality in solid organ or stem cell transplantation recipients and immunocompromised individuals such HIV patients [52, 53], where infection could result in interstitial pneumonia, retinitis, gastrointestinal tract complications like gastroenteritis, hepatitis, and graft failure [54]. Added to the previously mentioned hosts, HCMV infection poses a real burden in congenitally infected newborns with immature immune system, resulting possibly in deafness and neurodevelopmental delay [55]. HCMV exhibits two modes of viral infection: a lytic and a latent one [56]. The lytic phase is a highly regulated stage that ensures the production and the release of the new viral progeny outside the infected cells. This is followed by latency, a state characterized by a lifelong persistence in the host with the ability to reactivate under certain circumstances [53, 54]. During lytic infection, HCMV endures a well-regulated cascade of gene expression that starts with the expression of the immediate early viral genes [57] via the interaction of various cellular factors with the major immediate-early promoter (MIEP) [58]. This is followed by the expression of the early viral genes that play a role in the cellular modulation to favor viral replication [59] and later on by the late viral gene expression that ensures viral progeny assembly and release [60]. Recent studies have shown that epigenetic modifications play a role in the early productive infection events [61]. In fact, after viral entry, viral DNA rapidly becomes associated with histones, which makes it a vulnerable candidate to epigenetic modifications [62]. Such modifications usually result in a silent repressive state to viral gene expression as an intrinsic cellular defense mechanism. However, this repression is ultimately overcome, allowing the sequential expression cascade of lytic viral genes mentioned previously [63].

**Fig. 2:**
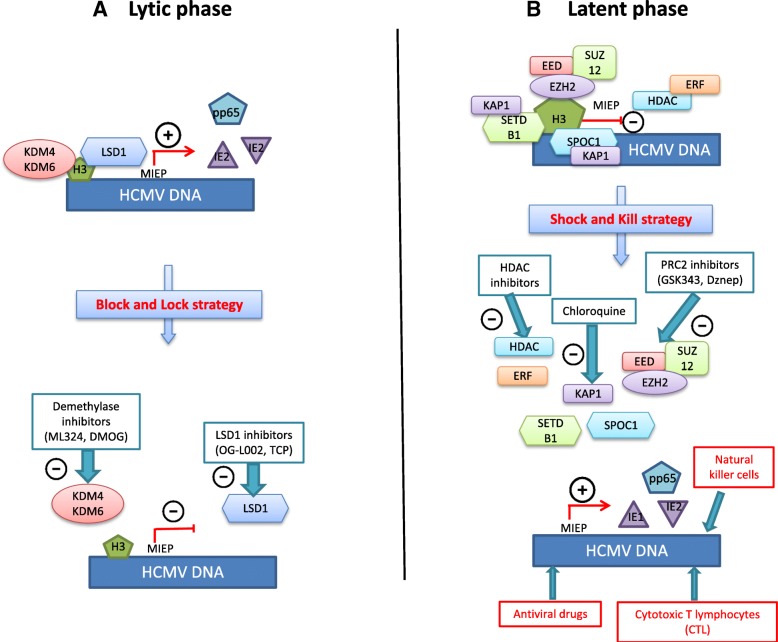
Schematic representation of the interplay between HCMV and epigenetic players in the context of lytic and latent infection. **a** During lytic infection, the repressive marks silencing the major immediate-early promoter (MIEP) are rapidly overcome, which results in the expression and transcription of the immediate early (IE) proteins. Histone demethylase (HDM) inhibitors can reverse and block viral activation at an early stage of infection, as well as during viral reactivation. **b** During latency, the repressive inhibition of the MIEP could be reversed by the polycomb complex 2 (PRC2) inhibitors or chloroquine, considered as latency reversal agents. The activated transcriptional program could purge the viral reservoirs (shock) and possibly achieve a sterilizing cure (kill) along with antivirals treatment. Alternatively, histone deacetylase (HDAC) inhibitors might induce a transient viral antigen expression, the latter being a target for pre-existing IE-specific cytotoxic T lymphocytes (CTL)

Although the few available antiviral drugs have granted chief advances in HCMV disease treatment and prophylaxis, their clinical applicability and utility confront several barriers [64]. First, resistance to antivirals is documented after prolonged use [65]. Added to this is the poor oral bioavailability and the dose-limited hematologic and renal toxicities reported with their use [66]. Moreover, they do not target the latent viral form in the host, which leaves the door open for viral shedding and transmission in saliva, urine, milk, vaginal secretions, and other bodily fluids [67]. It is worth to mention that the current antivirals used in HCMV management are ganciclovir, and its oral prodrug valganciclovir, cidofovir, and foscavir that target the viral DNA polymerase, in addition to fomivirsen, an antisense antiviral drug used in the treatment of CMV retinis [68], and the recently FDA-approved letermovir used to prevent viral infection following allogenic hematopoietic stem cell transplant [69]. Since those antivirals target the viral DNA replication step, the function and expression of the immediate early (IE) and early (E) HCMV genes during the early stages of infection are not blocked, paving the road to immunopathology and raise the risk of graft rejection [70]. Thus, this mandates and sheds the light on the urgent necessity of developing new antiviral drugs with novel mechanisms of action based on new potential viral or cellular targets. This is particularly conceivable with the enhanced understanding of HCMV molecular biology and the epigenetic mechanisms involved with its regulation (Fig. 2, Table 1).

### Reactivating HCMV from the latent phase the “shock and kill” paradigm

In contrast to the virus’ wide tropism during productive infection [71], several laboratories have pinpointed the myeloid lineage CD14+ monocytes and their CD34+ hematopoietic progenitor cells as important sites of HCMV latency in vivo [72]. While much is known about lytic infection, mechanisms of latency establishment and maintenance are still not fully elucidated [73]. This is due in part to difficulties of studying this process in vivo [74]. Indeed, extensive research showed that what was thought to be a quiescent state is in fact a highly active process characterized by the expression of a number of latency-associated viral genes [75]. Interestingly, a strong interplay exists between HCMV latency and epigenetic regulation through alterations of histones and other factors interacting directly or indirectly with the genome [76]. It has been shown that the removal of the repressive modifications associated with the latent phase can set in motion the lytic cycle and favor lytic gene expression [77]. The polycomb repressive complex 2 (PRC2) is a multi-subunit chromatin-modifying complex that plays a central role in regulating cellular differentiation, development, stem cell maintenance, and lineage specification [78, 79]. PRC2 complex is composed of the following core subunits: the embryonic ectoderm development (EED), the zinc finger protein suppressor of zeste 12 (SUZ12), and the enhancer of zeste homolog 2 (EZH2) that is responsible of the catalytic activity [80]. EZH2 protein is a histone methyltransferase (HMT) responsible for the tri-methylation of the lysine 27 residues of histone H3 (H3K27me3), which is generally associated with transcriptional silencing [81]. Several studies have linked HCMV latency to PRC2 activity [82–84]. In one study, chemical inhibitors of PRC2 were studied in THP1 monocytes and NT2D1 embryonal carcinoma cells, as models of HCMV quiescence. The methyltransferase inhibitor 3-deazaneplanocin A (DZnep) resulted in significant activation of the lytic transcriptional program, detected through the temporal regulated increase in viral transcript and antigen levels [85]. KAP1 (KRAB-associated protein 1) is a transcriptional co-repressor protein whose C-terminal effector end interacts with the H3K9me3-specific HMT SETDB1 (SET domain bifurcated 1) [86] and recruits it to the genome, which triggers H3K9 methylation and heterochromatin formation. It has been shown that during HCMV lytic infection, KAP1 is unable to repress transcription due to its suppression by mTOR-mediated phosphorylation. Pharmacological induction of KAP1 phosphorylation on serine 824 by the ATM activator chloroquine released HCMV from its latent state by blocking its ability to bind to SETDB1 and recruit it [87]. This approach could be added to the previously mentioned strategies to purge the HCMV latent reservoirs. Interestingly, survival time-associated PHD finger protein in ovarian cancer 1 (SPOC1), a recently identified restriction factor against HCMV, associates with the proximal enhancer region of the MIEP and promotes heterochromatin condensation possibly through the recruitment of corepressors that SPOC1 is known to interact with notably the previously mentioned KAP-1 and H3K9 methyltransferase [88]. On the other hand, Ets-2 repressor factor (ERF) is a cellular protein that physically interacts with the HCMV MIEP and functions as a transcriptional repressor of the latter by suppressing IE gene expression [89]. GST fusion assays showed a strong interaction between ERF and the N-terminus of HDAC1, a result that was further confirmed in vivo, suggesting that the physical interaction between ERF and HDAC1 could mediate repression of the MIEP [90]. Moreover, ying-yang 1 (YY1), a zinc finger DNA-binding protein and a multifunctional transcription factor [91], has been shown to repress the HCMV MIEP [92] partly by indirectly recruiting HDACs to the promoter via the nuclear protein SAP30, a component of the human HDAC complex [93]. In fact, forcing HCMV out of latency along with the conventional antiviral drug use in an attempt to eradicate it and establish a sterilizing cure is in correspondence with the “shock and kill” concept currently studied in HIV. Nevertheless, a better understanding of the molecular mechanisms involved in HCMV latency and reactivation could open future avenues for HCMV infection control.

### Repressing both HCMV lytic and reactivation phases by epigenetic-targeted therapy, “blocking and locking” the virus

HCMV lytic cycle and reactivation from latency are under the control of several epigenetic mechanisms. For example, histone demethylases (HDMs), due to their ability to remove the repressive marks, will promote productive infection. HDMs constitute a large family of more than 20 demethylases that are divided into two functional enzymatic families: the Lys-specific demethylase (LSD), also known as KDM1A and the Jumonji C (JMJC) protein families [94]. Two HDMs were studied in the context of HCMV lytic infection: KDM4 (JMJD2) and KDM6 (UTX/JMJD3), which demethylase histone H3-lysine 9 and lysine 27, respectively [95]. By using the JMJD2 demethylase inhibitor ML324, viral IE gene expression and viral yields were potently repressed, which could suggest that targeting these histone demethylases may potentially block viral gene expression and viral replication at a very early stage of infection and possibly abrogate it. In the same perspective, the LSD1 inhibitor OG-L002 repressed the expression of HCMV IE expression in HCMV-infected MRC5 cells [96]. In addition, the use of another LSD1 inhibitor, tranylcypromine (TCP), or alternatively the JMJD2 inhibitors, dimethyloxalylglycine (DMOG) or the previously mentioned ML324, resulted in a decrease in the expression of HCMV IE genes UL37, UL72, and US3 with a noted potent inhibition of IE gene expression with ML324 [97]. Thus, HDMs inhibitors could provide a therapeutic tool to target the initiation of infection or the spontaneous reactivation by blocking the viral cycle at an early stage, as a mimic for the proposed HIV “block and lock” strategy. This could be highly beneficial in the context of HCMV infection, as the expression or functions of viral IE and E gene products has shown their potential ability to elicit immuno-inflammatory responses that can lead to tissue rejection [98]. Not limited to inflammatory damage, some IE gene products can significantly interfere with important oncogenic signaling pathways and exhibit oncomodulatory properties, such as in glioblastoma cells [99]. In the context of oncomodulation, the PRC2 complex is associated with HCMV latency by the induction of transcriptional silencing [81–84]. HCMV-infected cells have been showed to exhibit enhanced expression of cellular oncogenic pathways, including c-Myc, c-Fos, c-Jun, Akt, and NF-κB [100–103]. This transcriptional activation will in turn lead to an increased expression of EZH2, resulting in an auto-amplifying loop. This oncomodulatory effect of HCMV infection could be targeted by PRC2 inhibitors and HDAC inhibitors that could both block cellular transformation and induce the activation of the viral lytic transcriptional program. This latter effect could allow through the expression of IE antigens the infected cells to be cleared by CMV-specific cytotoxic T cells (CTLs) [104].

## Epigenetic therapy, a general approach to cure viral infections?

Besides infection with HIV and HCMV, any viral infection might be potentially treated by new therapeutics targeting the epigenetic mechanisms (Table 1). We present below several examples of viral infections which could benefit from such new therapies.

### Hepatitis B virus

Hepatitis B virus (HBV) is a highly transmissible double-stranded DNA virus responsible of acute and chronic hepatitis B (CHB) in humans. Worldwide, about 240 million people are chronically infected, which could ultimately lead to liver fibrosis, cirrhosis, and the development of hepatocellular carcinoma (HCC) [105]. Despite the availability of several FDA-approved drugs like nucleos(t)ide analogs and pegylated interferon, clinical management remains problematic as cure is rarely achieved and the risk of resistance with long-term use and relapse after therapy discontinuation are common [106]. This is partly attributable to HBV persistence, despite treatment, as an episomal non-integrated covalently closed circular (ccc) DNA in the hepatocyte nucleus where it forms a highly stable minichromosome susceptible for epigenetic modifications [107]. Recently, as with the aforementioned viruses, the role of the epigenetic machinery in HBV persistence is gaining much attention [108]. Genome-wide maps of de novo infected HepG2 -NTCP1 cells, primary human hepatocytes (PHH), and HBV-infected liver tissue have shown that posttranslational modifications (PTM) that set in motion active transcriptional states are enriched at specific sites within the HBV covalently closed circular DNA (cccDNA) chromatin. Precisely, those PTM encompass high levels of H3K4me3, found at the transcription start site (TSS) of actively transcribed genes, as well as H3K27ac and H3K122ac, indicative of active gene enhancers [109]. This suggests that targeting epigenetic regulation could offer a new insight into new therapeutic approach to treat CHB. In fact, several options targeting epigenetic regulation are being studied with different desired end points: either a complete silencing of cccDNA or its complete elimination through reactivation and subsequent eradication. For instance, SIRT3, a class III HDAC, restricted HBV cccDNA transcription in PHH cells, possibly by increasing the recruitment of the HMT SUV39H1 and decreasing SETD1A recruitment, resulting in a marked increase of H3K9me3 and a decrease of H3K4me3 on cccDNA [110]. Moreover, treatment with the small molecule C646 that specifically inhibits p300/CBP, the histone acetyltransferases (HAT) for H3K27ac and H3K122ac reduced HBV transcription in a dose-dependent manner in the absence of measurable toxicity [111]. Likewise, PRMT5, a protein arginine methyltransferase 5, restricted HBV transcription and replication partly through regulation of symmetric dimethylation of arginine 3 on H4 on cccDNA exclusively [109]. In the same context, PRMT1, another arginine methyltransferase, is directly recruited to cccDNA, where its overexpression results in a 60% inhibition of HBV transcription in HepG2 cells. It is worthy to mention that this transcription inhibitory effect is limited to PRMT1 as PRMT3 overexpression did not affect transcription. Interestingly, PRMT1 was shown to interact with the regulatory hepatitis B virus X protein (HBx), which in turn inhibits PRMT1 methyltransferase activity [112]. HBx protein is a multifunctional regulatory protein that enhances HBV replication in vitro and in vivo [113] and affects numerous cellular processes including apoptosis [114], DNA repair mechanism [115], mitochondrial function [116], and cell signaling [117, 118]. Importantly, HBx could epigenetically influence cccDNA transcription through its recruitment onto the cccDNA minichromosome where it modulates the recruitment of chromatin-modifying enzymes such as the acetyltransferase p300 and HDACs including Sirt1 and HDAC1 [119]. HBx could also induce epigenetic aberrations that may lead to HBV-related HCC [120]. Those abnormalities include hypermethylation of several tumor suppressor genes, including—but not limited to—IGFBP-3 by DNMT3A1 and DNMT3A2 [121] and the E-cadherin promoter by DNMT1 [122]. Treatment with the DNMT inhibitor AZA restored the expression of the HBx-mediated epigenetically repressed secreted frizzled-related protein 1 (SFRP1), resulting in tumor growth inhibition and decreased aggressiveness in vitro and in vivo through negatively regulating the Wnt/β-catenin signaling pathway. This effect was further synergized by the use of the HDAC inhibitor trichostatin A (TSA) [123]. HBx-induced upregulation of SIRT2 expression promotes HBV replication in HepAD38 cells and enhances cell migration and invasion in the human hepatoma Huh7 cells, facilitating thus hepatocarcinogenesis [124]. The HBx protein upregulates the insulin-like growth factor 2 (IGF2) oncogene through hypomethylation of its promoter resulting in a poorer clinical outcome for HBV-related HCC patients [125]. Further understanding of the exact mechanisms of HBx-induced epigenetic alterations is highly needed, as those modifications could be used as biomarkers for the detection of early malignant transformation or as potential targets to treat persistent infection or HBV-related HCC. Nevertheless, for other epigenetic players such as DNMT, a critical balance in the context of HBV-related HCC should be maintained. Although DNMTs induce a decrease in the viral gene expression and replication [126], it could also result in silencing of tumor suppressor genes through DNA methylation, contributing thus to hepatocarcinogenesis [127]. On the other hand, treatment of HBV-transfected HuH7 cells with class I/II HDAC inhibitors, valproic acid (VPA) and TSA resulted in an increase in HBV transcripts, cytoplasmic accumulation of HBV replicative intermediates, and an increase in secreted HBV viral particles [128]. This was on controversy to a study that showed that some HDAC inhibitors like TSA and apicidin, a class I HDAC-specific inhibitor, suppressed cccDNA transcription in a duck hepatitis B virus (DHBV)-transfected chicken hepatoma cell line [129], which could be possibly due to some specificities or differences at the cellular level between the avian and the human cell model or at the viral level between the human and the duck virus.

### Hepatitis C virus

Infection with hepatitis C virus (HCV), a small enveloped RNA virus [130], is considered a health concern worldwide [131]. Although acute HCV infection can develop into a chronic condition with life-threatening complications including cirrhosis and HCC [132], recent antiviral treatments have highly improved the disease outcome. Even though previous treatment options were limited to interferon and ribavirin regimens, direct-acting antiviral (DAA) therapies based on HCV protease inhibitors have granted a definitive cure in more of 95% of patients [133]. Nevertheless, HCV-infected patients with genotype 3 represent up to 30% of all HCV infections worldwide and belong to the difficult-to-treat subgroup patients [134]. Therefore, epigenetic-targeted therapy could be a useful therapeutic tool not only for these HCV-infected difficult-to-treat patients, but also in HCV-HBV and HCV-HIV co-infected patients. Interestingly, recent studies are revealing a possible link between the molecular mechanisms of HCC carcinogenesis and host epigenetic alterations induced by HCV infection. The HDAC inhibitor SAHA suppressed HCV replication without affecting cell viability in Cellosaurus OR6 cell line. This suppression was linked to changes in gene expression through a SAHA-mediated increase in H3 acetylation levels of the promoter regions of several genes, resulting in an increased expression of osteopontin OPN [135]. Interestingly, OPN is a key cytokine that initiates Th1 immune response through regulating IL-12 and IL-10 cytokine expression [136]. Thus, SAHA-induced increased expression of OPN could possibly eliminate HCV by activating the Th1-type immune system. Correspondingly, tubastatin A, a selective inhibitor of HDAC6 suppressed HCV replication in HepG2 cells, along with α-tubulin hyperacetylation [137]. Although α-tubulin is known to be deacetylated by the histone deacetylase HDAC6 [138], the latter also controls the acetylation levels of other targets such as peroxiredoxins Prx1/2 [139] and the chaperone Hsp90 [140]. Thus, studying the effects of tubastatin A on those targets would be necessary to unveil the mechanism(s) by which this inhibitor is mediating its antiviral activity. Similarly, the HDAC3 inhibitor RGFP966 reduced viral replication in Huh7 cells and in in vivo model of humanized transgenic mice [141] with a downregulation in Apo-A1 expression, an indispensable protein for HCV infectivity maintenance [142], leading possibly to HCV secretion suppression. On the other hand, it has been shown that HCV infection could result in the DNA hypermethylation of some epigenetic markers [143]. For example, the methylation of the suppressor of cytokine signaling 1 (SOCS1), a negative regulator of the JAK/STAT pathway regarded as a tumor suppressor gene [144], was found to be positively associated with HCV infection status [145]. In the same context, the promoter of the tumor suppressor gene GADD45 (growth arrest and DNA damage-inducible gene 45) was detected to be hypermethylated in the context of HCV infection in mice transgenic for the entire HCV open reading frame, notwithstanding that the exact HCV-altered methylation mechanisms during infection remain to be explored [146]. The hypermethylation of GADD45 promoter by HCV downregulates GADD45 gene expression and interferes with its ability to block proliferation and tumorigenesis [147]. In addition, hepatitis C virus core protein has been linked to E-cadherin and p16 downregulation through upregulation of DNMT1 and DNMT3b [148, 149]. Thus DNMT inhibitors could constitute a novel approach for the treatment HCV-associated HCC. In this context, 5-Aza-C and 5-Aza-dC, two well-known DNMT inhibitors, significantly inhibited HCV infection. Interestingly, this effect is due not only to a decreased DNMT expression, but also through DNMT1 degradation [150]. It is worthy to mention that DNMTs expression could be varied between the different HCV genotypes 1b, 2a, 3a, 4h, and 5a. For example, DNMT3b mRNA is upregulated in genotype 1b HCV but not changed in genotypes 2a, 3a, 4h, and 5a [151]. Hence, the identification of HCV-induced epigenetic regulation that may actively participate in tumorigenesis and linking their prevalence to different HCV genotypes could possibly decipher new therapeutic targets for HCV infection and HCC management.

### Herpes simplex virus

Herpes simplex virus 1 (HSV-1), a double-stranded DNA virus [152], is a ubiquitous pathogen which infects more than 50% of the population in the USA and Europe [153]. As with other members of the *Herpesviridae* family, HSV-1 conserves its ability to remain latent in the sensory neurons of the trigeminal ganglion as a nucleosome-associated episome in the nucleus of the host cell [154], which makes it vulnerable to epigenetic posttranslational modifications. Not limited to latent phase, viral promoters and transcribed genes were shown to be associated with histones during lytic infection [155]. A transcriptional factor, the CCCTC binding factor CTCF extensively binds to HSV-1 DNA during lytic infection where it promotes HSV-1 lytic transcription. CTCF knockdown increased the repressive histone marks H3K27me3 and H3K9me3 and reduced viral transcription and virus yield [156]. In the same context, treatment with the protein methylation inhibitor, 5′-deoxy-5′-methylthioadenosine (MTA), reduced the level of H3K4me3 mediated by the methyltransferase Set1, along with a decrease in the transcription and replication of HSV-1 [157]. Interestingly, the transcriptional coactivator host cell factor-1 (HCF-1) was found to be involved with transcriptional activation through the recruitment of the histone methyltransferases Set1 and MLL1, leading consequently to H3K4 trimethylation [158]. Also, HCF-1 interacts with three sets of acetyltransferases: MOF/NSL [159], ATAC [160], and CLOCK [161]. In addition to multiple acetyltransferase complexes, HCF-1 induces transcriptional activation during the G1-to-S phase transition [162]. Not only essential to the expression of immediate-early genes and initiation of lytic infection, HCF-1 could be also involved in the reactivation process. This is demonstrated by HCF-1 translocation and recruitment to IE promoters upon ex vivo reactivation in trigeminal ganglia neurons [163]. Given the multiple complexes with which HCF-1 is associated, discerning the dynamic mechanistic process and the possible interactions between HCF-1 and various transcription factors, coactivators, and chromatin modulation components could define a new aspect of HSV-1 lytic infection and thus identify new possible targets to control it. In fact, epigenetic marks could be manipulated to reinforce latency and prevent reactivation cycles, or to purge the viral reservoirs. Treatment of the quiescently infected PC12 (QIF-PC12) cells, an in vitro accepted latency model for HSV, by HDAC inhibitors sodium butyrate and TSA resulted in the production of infectious progeny [164]. However, the end point of HDACs interaction with the viral genome is more complex than initially presumed. In fact, four HDACi, TSA, VPA, SAHA, and suberohydroxamic acid (SBHA), reduced the number of HSV-1 genome that initiate replication in human foreskin fibroblasts (HFF) cells and human female osteosarcoma (U2OS) cells [165]. This is possibly due to the HDACi-induced increase in the levels of some intrinsic immunity proteins know to exhibit antiviral immunity like promyelocytic leukemia (PML) bodies [166]. Same was shown with the nucleosome remodeler chromodomain helicase DNA-binding 3 protein (CHD3) that mediates repression of HSV genome upon infection. The CHD3 protein conserves its ability to identify and bind the repressive histone marks H3K27-trimethyl and H3K9-trimethyl, promoting the formation of heterochromatin [167, 168]. Surprisingly, the EZH2/1 inhibitors GSK126 and GSK343 suppressed productive viral lytic phase and decreased viral yields instead of inducing activation in vitro and in vivo [169]. This was attributable to the fact that treatment with those inhibitors enhanced cellular antiviral state by triggering antipathogen pathways. In the same context, other epigenetic players could negatively impact the reactivation of HSV-1 latent or quiescent infection. The JMJD2 inhibitors DMOG or ML342 significantly decreased the viral titers in trigeminal ganglia of HSV-1 latently infected mice through suppressing IE gene transcription and expression [97]. Attractively, the monoamine oxidase inhibitor (MAOi) tranylcypromine repressed HSV IE gene expression and genome replication in vivo, decreased the severity of a virus-induced encephalitis and corneal blindness in mouse models, and blocked viral reactivation in trigeminal ganglia [170, 171]. This is explained by the ability of MAOis to inhibit the LSD-1 mediated demethylation of lysine residues via a flavin-adenine-dinucleotide-dependent reaction [172, 173], resulting in the accumulation of repressive H3K9 chromatin marks at the IE promoters. Further studies are definitely needed to identify additional possible components and mechanisms involved in the epigenetic regulation of HSV-1 infection.

## Epstein-Barr virus

Epstein-Barr virus (EBV), or human herpesvirus 4 (HH4), is a DNA virus [174] that belongs to the *Herpesviridae* family, subfamily *Gammaherpesvirinae* [175]. Like other herpes viruses, EBV persists predominantly in the latently infected B lymphocytes as a covalently closed circular episome [176]. It has been shown that reactivation from latency is coupled to and initiated by expression of the viral BZLF1 gene [177]. During latency, BZLF1 promoter is silenced partly by the recruitment of repressive factors, such YY1 and the zinc finger E-box-binding factor (ZEB), that block the access of some transcriptional activators and ease the binding or function of repressive transcriptional co-factors like HDAC, maintaining thus a low levels of histone acetylation [178, 179]. Therefore, it is not surprising that HDAC inhibitors, like sodium butyrate, can reverse latency [180]. Not limited to low acetylation level, BZLF1 gene proximal promoter Zp is also silenced due to methylation. Treatment with DZNep, an H3K27me3 and H4K20me3 inhibitor, along with TSA stimulated BZLF1 expression level [181]. In fact, recognizing the mechanisms behind lytic reactivation is gaining an increased attention, due to the potential use of epigenetic inducing agents as sensitizers to conventional antivirals for the treatment of EBV-associated lymphomas [182]. Other than establishing a persistent latent infection, EBV infection is associated with several malignancies including Burkitt’s lymphoma (BL) [183], nasopharyngeal carcinoma (NPC) [184], Hodgkin’s lymphoma (HL) [185], and others [186, 187]. In the context of BL pathogenesis, regulation of Bim protein appears to be of high importance [188]. BIM is a member of the proapoptotic BH3-only family that acts as a cellular inducer of programmed cell death (PCD) by inactivating the function of the antiapoptotic BCL-2 and its homologs through binding or by directly activating the function of BAX and BAK [189]. During EBV latent infection, BIM expression is repressed, which increases the likelihood of B lymphomagenesis. Interestingly, latent EBV reduces acetylation of histones associated with Bim promoter and increases DNA methylation of the Bim promoter since the use of HDAC inhibitor TSA and the DNMT inhibitor AZA resulted in an increase in Bim mRNA and protein levels in EBV infected cells. In addition, the methylation of the CpG dinucleotides in the large CpG island located at the 5′ end of Bim in EBV-positive BL biopsies has been reported [190]. Besides Bim, p53 upregulated modulator of apoptosis (PUMA) is another proapoptotic BH3-only protein prone to regulation by EBV in the setting of Burkitt’s lymphoma. It was shown that EBV latency I genes, EBNA1, EBERs, or miR-BARTs, act cooperatively together to inhibit apoptosis by repressing PUMA [191]. For example, miR-BART5 binds PUMA in its 3′ untranslated region (UTR) which induces a decrease in PUMA transcription [192]. In addition, an in vitro model of continuously proliferating lymphoblastoid cell lines (LCLs) showed that EBV infection triggered CpG islands methylation of 40 tumor suppressor gene (TSG) including genes responsible of DNA-damage repair, cell cycle, and apoptosis regulation, resulting in a global transcriptional repression [193]. A comprehensive understanding of B cell reprogramming and epigenetic modifications could have important implications in the perception of EBV persistence and EBV-induced tumorigenesis, as well as potential therapeutic approaches in EBV-associated diseases. This could be exemplified by the fact that the epigenetic silencing through hypermethylation and deacetylation of the previously mentioned BIM gene is correlated with chemotherapeutic resistance (especially to doxorubicin) and lower complete remission rates in Burkitt lymphoma/leukemia. Interestingly, BIM could be re-expressed by the use of the HDAC inhibitor vorinostat, in a xenograft mouse model, resulting in cells sensitization to doxorubicin and cyclophosphamide, as evidenced by the increased survival rate [194].

## Epigenetic players in viral infections: filling the gap between basic research and clinical application

Powered by the supportive results in cell cultures and mouse models, epigenetic drug candidates are recently being clinically evaluated as potential antiviral drugs. In this section, some completed and ongoing clinical trials are cited, as an attempt to present preliminary data about the use of epigenetic drugs to manipulate viral infections or viral infection-related malignancies (Table 2). As most trials are being published recently, analyzing toxicity, schedules, doses, and measuring clinical response stand up as the main aim. In the setting of HIV infection, several HDAC inhibitors were tested as a combination with antiretroviral therapy: panobinostat (NCT01680094), vorinostat (NCT01319383), and romidepsin (NCT02092116, NCT01933594), in addition to VPA (NCT00289952). Although with varying degrees, all HDAC inhibitors showed an increase in viral transcription with no significant effect on the size of the HIV-1 functional reservoir, as no inhibitor has demonstrated complete clearance of latent infection [195–198]. This is possibly attributable to incomplete latency reversal or insufficient clearance of latency-reactivated cells. It is suggested that the clearance of HIV latent reservoir could be enhanced by adding immune enhancement treatments, such as the immunomodulatory cytokine interferon-alpha2a with panobinostat (NCT02471430); the therapeutic vaccine MVA.HIVconsv (NCT02616874); the bNAb-based therapeutic HIV vaccine 3BNC117 (NCT03041012) with romidepsin; alternatively, the autologous dendritic cell vaccine AGS 004 (NCT02616874); or disulfiram (NCT03198559) with vorinostat, as disulfiram was shown to reactivate latent HIV-1 in a primary CD4+ T cell model [199]. Importantly, caution should be engaged as the increased efficacy implicated by those combinations could be complemented with adverse effects not noted with the used of HDAC inhibitors alone. Not limited to HIV infection, some HDAC inhibitors have been tested in the context of viral-induced malignancies. Treatment with belinostat demonstrated tumor stabilization in unresectable hepatocellular carcinoma (NCT00321594) [200]. In addition, mocetinostat (MGCD0103) showed promising disease control in patients with relapsed classical Hodgkin lymphoma (NCT00358982) [201]. Tractinostat (VRx-3996) in combination with valganciclovir is currently under investigation in EBV-associated lymphoid malignancies (NCT03397706). Interestingly, HDAC inhibitors could induce EBV lytic-phase gene expression and act as sensitizers to antivirals for the treatment of EBV-associated lymphomas [202] as in the case of arginine butyrate and ganciclovir [203]. Another epigenetic player, the DNMT inhibitor azacitidine, was shown to reverse the dense CpG methylation and potentially triggering gene re-expression in patients with EBV-positive tumors [204]. Although those inhibitors might be future promising candidates for inclusion in the current therapeutic management, caution should be taken as they could reactivate some latent DNA viruses like HBV or EBV in the setting of other conditions treatment [205]. For instance, the use of SAHA or TSA aggravated the severity of myocarditis induced by coxsackievirus B3 (CVB3) through CVB3-induced myocardial apoptosis [206]. Thus, a careful assessment before the use of HDAC inhibitors is highly needed.

**Table 2:** Clinical trials of histone deacetylase inhibitors in viral infections and viral-associated malignancies

Drug	Combination	Indication	Clinical result	Study phase and status	Trial*	Reference
Panobinostat	Antiretroviral therapy	HIV infection	-Increase in unspliced HIV RNA -No reduction in integrated HIV DNA -Safe, well tolerated	Phases I–II	NCT01680094	[195]
	-Antiretroviral therapy -Interferon-alpha2a	HIV infection	Ongoing	Phases I–II	NCT02471430	
Vorinostat	Antiretroviral therapy	HIV infection	-Increase in cell-associated HIV RNA with no effective depletion of persistent HIV reservoir -Safe, well tolerated	Phases I–II	NCT01319383	[196]
	-Antiretroviral therapy -Autologous dendritic cell vaccine (AGS 004)	HIV infection	No published results yet	Phase I	NCT02707900	
	-Antiretroviral therapy -Disulfiram	HIV infection	Suspended	Phases I–II	NCT03198559	
Romidepsin	Antiretroviral therapy	HIV infection	-Increase in cell-associated un-spliced HIV-1 RNA -No effect on the number of HIV-specific T cells -No severe adverse events	Phases I–II	NTC02092116	[197]
	Antiretroviral therapy	HIV infection	No published results yet	Phases I–II	NCT01933594	
	MVA.HIVconsv vaccine	HIV infection	No published results yet	Phase I	NCT02616874	
	Broadly neutralizing antibody (3BNC117)	HIV infection	Ongoing	Phase II	NCT03041012	
Valproic acid	Antiretroviral therapy	HIV infection	No significant reductions in the frequency of CD4+ T cells harboring replication-competent HIV	Phase II	NCT00289952	[198]
Belinostat	None	Unresectable hepatocellular carcinoma	-Tumor stabilization -Well tolerated	Phases I–II	NCT00321594	[200]
Mocetinostat	None	Relapsed and refractory classical Hodgkin lymphoma	-Decrease in tumor measurements -Grade 3 and 4 adverse events (neutropenia and pneumonia)	Phase II	NCT00358982	[201]
Tractinostat	Valganciclovir	EBV-associated lymphoid malignancies	Ongoing	Phase Ib/II	NCT03397706	

*Retrieved from Clinicaltrials.gov

## Conclusion and future perspectives

Over the past years, epigenetic studies have revealed novel principles and profoundly broadened our knowledge about the interplay between viruses, cellular transcription factors, histones, and nonhistones modifying enzymes. As most of those modifications are reversible, manipulating this complex machinery could have a critical role in determining an active lytic or latent viral infection and subsequent viral reactivation from latency. This diverts the end goal to permanently silence the virus in latent reservoirs so that the possibility of reactivation is diminished, or to eradicate it through purging the viral reservoirs after reactivating it. Advantageously, and in contrast to the conventional antivirals, it is hypothesized that the emergence of resistant strains is minimized, as those chromatin modulation components target the host, rather than viral-encoded factors. Furthermore, epigenetic therapy could exemplify the “two birds, one stone” concept in the scenario of viral co-infection, e.g., with HCMV-HSV, HIV-HCV, and HIV-HBV. However, this tremendous array for new targets is a double-edged sword. In fact, the available epigenetics therapies lack specificity, which raises questions about their cytotoxic side effects due to unintended global epigenetic modifications and complicates the achievement of a therapeutic index within the acceptable toxicity levels. Intriguingly, designing and testing target-specific inhibitors (specific HDAC inhibitors rather than pan-inhibitors for example) could improve therapeutic outcomes through dropping the off-target undesired effects. This could be partly achieved through studying structure-activity relationship (SAR) to select a potent and selective compound for further mechanistic studies. In addition, advancement in epigenetic analysis tools such as epigenome microarray and combining chromatin immune-precipitation (Chip) to next-generation sequencing (NGS) could provide a useful tool to decipher the multiprotein complexes involved in the epigenetic control of viral infections. In addition, addressing the role of the less studied post-translational modifications such as phosphorylation or sumoylation can shed light on new aspects of the dynamic host-viral interplay. Altogether, new therapeutic approaches are actively needed to fight viral infections and drugs targeting epigenetic players could lead to major therapeutic breakthroughs in the future.

## Abbreviations

5-Aza: 5-Azacytidine
BL: Burkitt’s lymphoma
cART: Combination antiretroviral therapy
cccDNA: Covalently closed circular DNA
CHB: Chronic hepatitis B
CTL: Cytotoxic T cells
CVB3: Coxsackievirus B3
DAA: Direct-acting antiviral
dCA: Didehydro-cortistatin A
DHBV: Duck hepatitis B virus
DMOG: Dimethyloxalylglycine
DNMT: DNA methyltransferase
EBV: Epstein-Barr virus
EED: Embryonic ectoderm development
ERF: Ets-2 repressor factor
EZH2: Enhancer of zeste homolog 2
GADD45: Growth arrest and DNA damage-inducible gene 45
HAART: Highly active antiretroviral therapy
HAT: Histone acetyltransferases
HBV: Hepatitis B virus
HBx: Hepatitis B virus X protein
HCC: Hepatocellular carcinoma
HCF-1: Host cell factor-1
HCMV: Human cytomegalovirus
HCV: Hepatitis C virus
HDAC: Histone deacetylase
HDM: Histone demethylase
HHV4: Human herpesvirus 4
HHV5: Human herpesvirus 5
HIV: Human immunodeficiency virus
HL: Hodgkin’s lymphoma
HMT: Histone methyltransferase
HSV-1: Herpes simplex virus 1
IE: Immediate early
IGF2: Insulin-like growth factor 2
JMJC: Jumonji C
KAP1: KRAB-associated protein 1
LCLs: Lymphoblastoid cell lines
LRA: Latency reversing agent
LSD: Lys-specific demethylase
LTR: Long terminal repeat
MAOi: Monoamine oxidase inhibitor
MIEP: Major immediate-early promoter
MTA: Methylthioadenosine
NPC: Nasopharyngeal carcinoma
PCD: Programmed cell death
PHH: Primary human hepatocytes
PKC: Protein kinase C
PRC2: Polycomb repressive complex 2
P-TEFb: Positive transcription elongation factor B
PTM: Posttranslational modifications
PUMA: p53 upregulated modulator of apoptosis
SAHA: Suberanilohydroxamic acid
SBHA: Suberohydroxamic acid
SETDB1: SET domain bifurcated 1
SFRP1: Secreted frizzled-related protein 1
SOCS1: Suppressor of cytokine signaling 1
SPOC1: Survival time-associated PHD finger protein in ovarian cancer 1
SUZ12: Suppressor of zeste 12
Tat: Transactivator of transcription
TCP: Tranylcypromine
TNF: Tumor necrosis factor
TPA: 12-O-Tetradecanoylphorbol-13-acetate
TSG: Tumor suppressor gene
TSS: Transcription start site
UTR: Untranslated region
VPA: Valproic acid
YY1: Ying-yang 1
